# Clinicopathologic features and prognostic factors in early- versus late-onset colorectal cancer: a stage-stratified retrospective surgical cohort from the Mekong Delta, Vietnam

**DOI:** 10.1590/0102-672020260000038e1967

**Published:** 2026-08-31

**Authors:** Kiet Nguyen LE GIA, Nang Van PHAM, Tuan Van NGUYEN, Doi Van MAI, Hien Van NGUYEN, Huan Hoang LAM, Tuan Thanh TRAN, Phu Thien Diep DUONG, Trinh Vo Thi ANH

**Affiliations:** 1 Can Tho University of Medicine and Pharmacy, Department of General Surgery - Can Tho City, Vietnam.; 2 Vinmec Can Tho Hospital - Can Tho City, Vietnam.; 3 Can Tho University of Medicine and Pharmacy, Faculty of Medicine - Can Tho City, Vietnam.

**Keywords:** Colorectal neoplasms, Age of onset, Prognosis, Operative surgical procedures, Laparoscopy, Neoplasias colorretais, Idade de início, Prognóstico, Procedimentos cirúrgicos operatórios, Laparoscopia

## Abstract

**Background:**

Early-onset colorectal cancer (EOCRC), defined as diagnosis before 50 years of age, has increased in incidence globally, but its clinicopathologic profile and prognostic significance remain incompletely characterized, particularly in Southeast Asian populations with limited access to organized screening.

**Aims:**

To compare clinicopathologic characteristics and survival outcomes between EOCRC and late-onset colorectal cancer (LOCRC) following curative resection, and to identify independent prognostic determinants in a regional Vietnamese surgical cohort.

**Methods:**

This retrospective cohort study included 486 patients with stage I-III colorectal adenocarcinoma who underwent curative laparoscopic resection at a tertiary center in the Mekong Delta between 2016 and 2022.

**Results:**

EOCRC (184 patients, 37.9%) was associated with higher rates of poor differentiation, mucinous or signet-ring cell histology, lymphovascular invasion, and perineural invasion compared with LOCRC (all p<0.05). Overall survival (OS), cancer-specific survival (CSS), and recurrence-free survival (RFS) did not differ significantly between groups. In the overall cohort (log-rank p=0.72, 0.15, and 0.96, respectively). On multivariable analysis, postoperative complications were the dominant independent prognostic factor for OS (hazard ratio [HZ] 8.28; 95% confidence interval [CI] 4.87-14.09) and RFS (HZ 4.81; 95%CI 3.03-7.65), whereas age at onset was not independently associated with either outcome. Stage-stratified curves suggested a less favorable survival pattern among patients with stage III EOCRC, particularly for CSS and RFS.

**Conclusions:**

Despite a more aggressive histopathologic profile, EOCRC showed comparable survival to LOCRC in the overall cohort. Stage-stratified curves suggested a less favorable survival pattern among patients with stage III EOCRC, while postoperative complications represented the dominant modifiable determinant of outcome.

## ARTICLE HIGHLIGHTS

• Early-onset colorectal cancer was associated with more aggressive histopathologic features than late-onset colorectal cancer.

• Overall survival, cancer-specific survival, and recurrence-free survival were comparable between early- and late-onset colorectal cancer in the overall cohort.

• Stage-stratified curves suggested a less favorable survival pattern among patients with stage III early-onset colorectal cancer, particularly for cancer-specific and recurrence-free survival.

• Postoperative complications were strongly associated with worse overall and recurrence-free survival after curative colorectal cancer surgery.

## Summary

**CENTRAL MESSAGE:**

Early-onset colorectal cancer showed more aggressive histopathologic features than late-onset colorectal cancer, but overall survival outcomes were comparable in the entire cohort. Stage-stratified curves suggested a less favorable survival pattern among patients with stage III early-onset colorectal cancer, particularly for cancer-specific and recurrence-free survival. Postoperative complications were strongly associated with worse long-term outcomes.

**PERSPECTIVES:**

This study provides real-world surgical oncology data from the Mekong Delta, Vietnam, a Southeast Asian region underrepresented in colorectal cancer research. The findings suggest that younger patients with colorectal cancer may present with more aggressive tumor biology, while long-term outcomes are strongly influenced by perioperative complications. Improving perioperative safety and strengthening early diagnostic strategies for symptomatic younger adults may help improve colorectal cancer outcomes in similar regional settings.

## VISUAL ABSTRACT

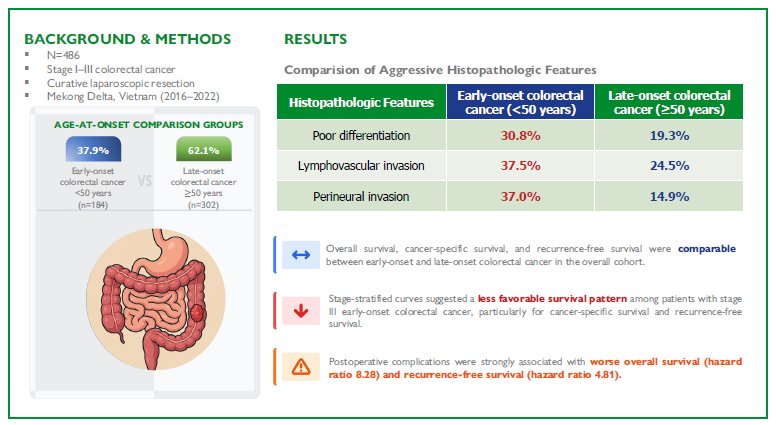

## RESUMO

**Racional:**

O câncer colorretal de início precoce (CCIP), definido quanto ao diagnóstico antes dos 50 anos de idade, apresenta aumento da incidência global. Contudo, seu perfil clinicopatológico e impacto prognóstico permanecem pouco caracterizados, especialmente em populações do Sudeste Asiático com acesso limitado ao rastreamento organizado.

**Objetivos:**

Comparar características clinicopatológicas e desfechos de sobrevida entre CCIP e câncer colorretal de início tardio (CCIT) após ressecção curativa, e identificar determinantes prognósticos independentes em uma coorte cirúrgica regional vietnamita.

**Métodos:**

Este estudo de coorte retrospectivo incluiu 486 pacientes com adenocarcinoma colorretal (estádios I-III) submetidos à ressecção laparoscópica curativa em um centro terciário no Delta do Mekong entre 2016 e 2022). A sobrevida foi analisada por estimativas de Kaplan-Meier e regressão de Cox multivariável.

**Resultados:**

O CCIP (184 pacientes; 37,9%) associou-se significativamente a menor grau de diferenciação, histologia mucinosa ou de células em anel de sinete, e invasão linfovascular e perineural em relação ao CCIT (p<0,05). A sobrevida global (SG), a sobrevida específica por câncer (SEC) e a sobrevida livre de recorrência (SLR) não diferiram entre os grupos na coorte geral (*log-rank* p=0,72; 0,15; e 0,96, respectivamente). Na análise multivariável, as complicações pós-operatórias foram o principal fator prognóstico independente para SG (razão de risco [HR] 8,28; intervalo de confiança [IC] de 95% 4,87-14,09) e SLR (HR 4,81; IC95% 3,03-7,65), enquanto a idade ao diagnóstico não afetou os desfechos independentemente. A análise estratificada por estádio sugeriu um padrão de sobrevida menos favorável entre pacientes com CCIP em estádio III, particularmente para a SEC e a SLR.

**Conclusões:**

Embora o CCIP exiba um perfil histopatológico mais agressivo, sua sobrevida na coorte geral é comparável ao CCIT. As curvas estratificadas por estádio sugeriram um padrão de sobrevida menos favorável entre pacientes com CCIP em estádio III, enquanto as complicações pós-operatórias representaram um determinante modificável importante do desfecho.

## INTRODUCTION

Colorectal cancer (CRC) remains a major cause of cancer-related morbidity and mortality worldwide^7^ ^,^ ^8^. Althoughscreening and treatment have improved outcomes among older adults, the incidence of CRC in individuals younger than 50 years continues to increase^4^ ^,^ ^14^ ^,^ ^15^ ^,^ ^19^. Early-onset colorectal cancer (EOCRC) has therefore become an important clinical and public health concern. Whether EOCRC differs from late-onset colorectal cancer (LOCRC) in tumor characteristics, stage at presentation, and long-term outcomes remains uncertain^1^ ^,^ ^4^ ^,^ ^13^.

Studies have suggested that EOCRC is more often associated with adverse histopathologic features, including poor differentiation, lymphovascular invasion, and perineural invasion^4^ ^,^ ^14^ ^,^ ^20^. However, the prognostic significance of these features remains unclear^4^ ^,^ ^6^. Some cohorts have reported worse outcomes in younger patients, whereas others have found no survival difference after adjustment for disease stage and treatment-related factors^1^ ^,^ ^14^. It also remains uncertain whether age at onset is independently associated with prognosis or whether prognostic differences emerge only after stratification by pathologic stage^4^ ^,^ ^8^ ^,^ ^20^.

This question is relevant in Vietnam and other Southeast Asian settings, where organized population-based CRC screening is not widely implemented and younger patients may present only after symptom onset^17^. Data on EOCRC from the Mekong Delta remain limited, although delayed presentation, referral patterns, and differences in access to care may influence clinicopathologic characteristics and outcomes^4^ ^,^ ^19^. A tertiary surgical cohort from this setting may therefore provide clinically relevant data from a population underrepresented in the international literature^17^.

A retrospective cohort study was conducted of patients with stage I-III colorectal adenocarcinoma who underwent curative resection at a tertiary hospital in the Mekong Delta, Vietnam. The objectives were to compare clinicopathologic characteristics between EOCRC and LOCRC, evaluate overall survival, recurrence-free survival, and cancer-specific survival, and assess whether age at onset, postoperative complications, and disease stage were independently associated with these outcomes, including in stage-stratified analyses.

## METHODS

### Study design and setting

This was a retrospective cohort study of patients with colorectal adenocarcinoma who underwent curative laparoscopic resection at the Department of General Surgery, Can Tho University of Medicine and Pharmacy Hospital, between January 2016 and December 2022. The study was approved by the Institutional Ethics Committee of Can Tho University of Medicine and Pharmacy (approval number: 26.003/PCT-HÐÐÐ). Written informed consent was waived owing to the retrospective nature of the study. All procedures were conducted in accordance with the Declaration of Helsinki.

### Patient selection

Patients were eligible if they had a histologically confirmed diagnosis of colorectal adenocarcinoma, underwent elective curative-intent resection, and had pathologically confirmed stage I-III disease according to the American Joint Committee on Cancer (AJCC) 8th edition staging system. Patients were excluded if they had stage IV disease (distant metastasis) at the time of surgery, underwent emergency surgery, had synchronous or metachronous colorectal malignancies, had a prior history of CRC or hereditary CRC syndrome (including Lynch syndrome or familial adenomatous polyposis), had incomplete clinicopathologic or follow-up data, or underwent non-curative (R1 or R2) resection.

### Study groups

Patients were classified into two groups according to age at diagnosis: EOCRC, defined as diagnosis before 50 years of age, and LOCRC, defined as diagnosis at 50 years of age or older. This threshold was prespecified based on the established epidemiological definition of EOCRC used in international literature.

### Data collection

Clinical, pathologic, operative, and follow-up data were extracted from the hospital’s electronic medical records and surgical pathology database. Extracted variables included patient demographics (age, sex), presenting symptoms, American Society of Anesthesiologists (ASA) physical status classification, tumor location (right colon, left colon, or rectum), AJCC pathologic stage, histologic subtype (conventional adenocarcinoma *vs*. mucinous or signet-ring cell carcinoma), tumor differentiation grade, lymphovascular invasion, perineural invasion, postoperative complications, receipt of adjuvant chemotherapy, and follow-up status.

Tumor location was defined as right colon (cecum to transverse colon), left colon (splenic flexure to sigmoid colon), and rectum. Postoperative complications were defined as any deviation from the normal postoperative course occurring within 30 days of surgery, classified according to the Clavien-Dindo system; major complications were defined as Clavien-Dindo grade III or higher.

Adjuvant chemotherapy decisions were made by a multidisciplinary team according to institutional protocols aligned with national guidelines and were administered primarily to patients with stage III disease and to selected patients with high-risk stage II disease (defined as T4 stage, fewer than 12 lymph nodes examined, lymphovascular invasion, perineural invasion, or poorly differentiated histology).

### Outcomes

The primary outcomes were overall survival (OS), cancer-specific survival (CSS), and recurrence-free survival (RFS). OS was calculated from the date of surgery to the date of death from any cause or last follow-up. CSS was calculated from the date of surgery to the date of death attributable to CRC. RFS was calculated from the date of surgery to the date of documented radiologic or pathologic recurrence, death from any cause, or last follow-up. Patients alive and without evidence of recurrence at the time of last follow-up were censored.

### Follow-up and recurrence definition

Follow-up was conducted through scheduled outpatient visits at three-month intervals for the first two years and at six-month intervals thereafter. At each visit, patients underwent clinical assessment and serum carcinoembryonic antigen measurement. Abdominal ultrasonography was performed at every follow-up visit as a first-line imaging modality for the detection of hepatic and intra-abdominal recurrence. Contrast-enhanced computed tomography of the chest, abdomen, and pelvis was performed every six months for the first two years and annually thereafter, or earlier if clinically indicated. For patients with rectal cancer, pelvic magnetic resonance imaging was additionally performed at six-month intervals for the first two years postoperatively to evaluate locoregional pelvic recurrence. Colonoscopy was performed at one year postoperatively and every three to five years subsequently, or earlier if clinically indicated. The last follow-up date was October 31, 2025.

Recurrence was defined as any radiologically or pathologically confirmed evidence of tumor recurrence following an initial disease-free interval after curative resection. Recurrence was classified as locoregional or distant. Locoregional recurrence included anastomotic recurrence, mesorectal or pelvic recurrence, and regional lymph node recurrence within the operative field. Distant recurrence included hepatic metastases, pulmonary metastases, peritoneal carcinomatosis, and distant lymph node or other organ metastases. Radiologic diagnosis of recurrence required new or enlarging lesions on computed tomography, abdominal ultrasonography, or pelvic magnetic resonance imaging consistent with metastatic or recurrent disease, confirmed by at least two independent imaging assessments or by histopathologic confirmation whenever feasible. Elevation of serum carcinoembryonic antigen alone, without corresponding imaging evidence, was not considered sufficient to define recurrence.

### Statistical analysis

Categorical variables were compared between groups using the Pearson ꭓ^2^ test or Fisher’s exact test, as appropriate. Continuous variables were summarized as median (interquartile range [IQR]) and compared using the Student’s *t*-test or the Mann-Whitney U test, depending on distributional assumptions assessed by the Shapiro-Wilk test.

Survival curves were constructed using the Kaplan-Meier method and compared between groups using the log-rank test. Univariable Cox proportional hazards regression was used to estimate hazard ratios (HRs) and 95% confidence intervals (CIs) for each candidate prognostic variable. Variables with p<0.10 on univariable analysis were entered into a multivariable Cox regression model to identify independent prognostic factors for OS and RFS. The proportional hazards assumption was verified using Schoenfeld residuals. Stage-stratified analyses were performed by grouping patients into four subgroups (EOCRC stage I-II, LOCRC stage I-II, EOCRC stage III, and LOCRC stage III) and comparing survival outcomes across subgroups using the log-rank test.

All statistical analyses were performed using Statistical Packae for the Social Sciences (SPSS) version 26.0 (IBM Corp., Armonk, NY, USA) and R software version 4.3.0 (R Foundation for Statistical Computing, Vienna, Austria). A two-tailed p<0.05 was considered statistically significant.

## RESULTS

A total of 486 patients underwent curative laparoscopic resection for stage I-III colorectal adenocarcinoma at a single center between January 2016 and December 2022. Their median [IQR] age was 57 (45-68) years; 273 (56.2%) were male; 184 (37.9%) had EOCRC, and 302 (62.1%) had LOCRC. The median (IQR) follow-up was 47 (39-82) months. During follow-up, 60 all-cause deaths, 43 cancer-specific deaths, and 93 recurrences were documented.

### Patient characteristics

There was no significant difference in gender distribution between the two groups (p=0.51). Baseline physical status differed markedly between groups: ASA class I was recorded in 94.6% of EOCRC patients *vs*. 7.0% of LOCRC patients, whereas ASA class II predominated among LOCRC (84.8% *vs*. 5.4%; p<0.001). Regarding clinical presentation, hematochezia was significantly more frequent in the EOCRC group (36.4% *vs*. 21.2%; p<0.001), while diarrhea was more common among LOCRC patients (10.6% *vs*. 4.9%; p=0.03).

Regarding tumor characteristics, right-sided colon cancer was more prevalent in the EOCRC group (26.6% *vs*. 15.6%), whereas rectal cancer was more common in the LOCRC group (55.6% *vs*. 44.6%; p=0.007) (Table 1). LOCRC patients had a higher proportion of stage III disease (73.5% *vs*. 56.0%), whereas EOCRC patients more frequently presented with stage II disease (37.5% *vs*. 22.5%; p<0.001). Histopathologically, EOCRC cases exhibited more aggressive features, including a higher rate of poor differentiation (30.8% *vs*. 19.3%; p=0.016), mucinous or signet-ring cell histology (15.8% *vs*. 9.6%; p=0.042), lymphovascular invasion (37.5% *vs*. 24.5%; p=0.003), and perineural invasion (37.0% *vs*. 14.9%; p<0.001) (Table 1).

**Table 1.** Baseline clinicopathologic characteristics of the study population stratified by age at onset.

Characteristic	Cohort (n=486) n (%)	EOCRC (n=184) n (%)	LOCRC (n=302) n (%)	p-value
Sex				
Male	273 (56.2)	107 (58.2)	166 (55.0)	0.510*
Female	213 (43.8)	77 (41.8)	136 (45.0)	
Clinical presentation				
Abdominal pain	55 (11.3)	19 (10.3)	36 (11.9)	0.659*
Weight loss	51 (10.5)	22 (12.0)	29 (9.6)	0.447*
Hematochezia	131 (27.0)	67 (36.4)	64 (21.2)	<0.001*
Constipation	45 (9.3)	15 (8.2)	30 (9.9)	0.525*
Diarrhea	41 (8.4)	9 (4.9)	32 (10.6)	0.029*
Tenesmus	50 (10.3)	25 (13.6)	25 (8.3)	0.066*
ASA physical status				
I	195 (40.1)	174 (94.6)	21 (7.0)	<0.001^†^
II	266 (54.7)	10 (5.4)	256 (84.8)	
III	25 (5.1)	0	25 (8.3)	
Tumor location				
Right colon	96 (19.8)	49 (26.6)	47 (15.6)	0.007*
Left colon	140 (28.8)	53 (28.8)	87 (28.8)	
Rectum	250 (51.4)	82 (44.6)	168 (55.6)	
AJCC stage				
I	24 (4.9)	12 (6.5)	12 (4.0)	<0.001*
II	137 (28.2)	69 (37.5)	68 (22.5)	
III	325 (66.9)	103 (56.0)	222 (73.5)	
Mucinous/signet-ring cell	58 (15.8)	29 (9.6)	29 (10.3)	0.042*
Differentiation				
Well differentiated	22 (4.6)	8 (4.4)	14 (4.7)	0.016*
Moderately differentiated	346 (71.8)	118 (64.8)	228 (76.0)	
Poorly differentiated	114 (23.7)	56 (30.8)	58 (19.3)	
Lymphovascular invasion				
No	343 (70.6)	115 (62.5)	228 (75.5)	0.003*
Yes	143 (29.4)	69 (37.5)	74 (24.5)	
Perineural invasion				
No	373 (76.7)	116 (63.0)	257 (85.1)	<0.001*
Yes	113 (23.3)	68 (37.0)	45 (14.9)	
Adjuvant chemotherapy	319 (65.6)	144 (78.3)	175 (57.9)	<0.001*

EOCRC: early-onset colorectal cancer (<50 years); LOCRC: late-onset colorectal cancer (≥50 years); ASA: American Society of Anesthesiologists; AJCC: American Joint Committee on Cancer.

*Pearson’s ꭓ^2^ test; ^†^Fisher’s exact test. (Data on differentiation were missing for two patients in the early-onset colorectal cancer group and two patients in the late-onset colorectal cancer group).

### Prognostic factor analysis

On multivariable analysis (Table 2), postoperative complications were independently associated with increased risk of death (HR 8.28; 95%CI 4.87-14.09; p<0.001) and recurrence (HR 4.81; 95%CI 3.03-7.65; p<0.001). Tumor location was associated with OS (overall p=0.02). Lymphovascular invasion, perineural invasion, and AJCC stage were not independently associated with OS or RFS after multivariable adjustment. Age at onset, sex, and adjuvant chemotherapy were not associated with OS or RFS in univariable analysis and were therefore not entered into the multivariable model.

**Table 2.** Univariable and multivariable Cox regression analyses for overall survival and recurrence-free survival.

Variable	Univariable OS HR (95%CI)	p-value	Multivariable OS HR (95%CI)	p-value	Univariable RFS HR (95%CI)	p-value	Multivariable RFS HR (95%CI)	p-value
Age, years								
<50	1.00 (Ref.)	0.73	-		1.00 (Ref.)	0.96	-	
≥50	1.10 (0.65-1.86)		-		0.99 (0.65-1.50)		-	
Sex								
Male	1.00 (Ref.)	0.19	-		1.00 (Ref.)	0.47	-	
Female	0.70 (0.41-1.19)		-		0.86 (0.57-1.30)		-	
Tumor location								
Right colon	1.00 (Ref.)	0.082	1.00 (Ref.)	0.02	1.00 (Ref.)	0.75	-	
Left colon	1.50 (0.74-3.04)		1.21 (0.59-2.49)		1.02 (0.57-1.82)		-	
Rectum	0.79 (0.39-1.62)		0.55 (0.27-1.13)		0.87 (0.51-1.48)		-	
Overall complication								
No	1.00 (Ref.)	<0.001	1.00 (Ref.)	<0.001	1.00 (Ref.)	<0.001	1.00 (Ref.)	<0.001
Y es	8.06 (4.80-13.53)		8.28 (4.87-14.09)		4.72 (2.98-7.47)		4.81 (3.03-7.65)	
Major complications								
No	1.00 (Ref.)	0.29	-		1.00 (Ref.)	0.87	-	
Yes	1.74 (0.63-4.79)		-		1.09 (0.40-2.96)		-	
Lymphovascular invasion								
No	1.00 (Ref.)	<0.001	1.00 (Ref.)	0.73	1.00 (Ref.)	<0.001	1.00 (Ref.)	0.34
Yes	3.41 (2.05-5.69)		1.35 (0.25-7.34)		2.47 (1.65-3.72)		1.61 (0.60-4.29)	
Perineural invasion								
No	1.00 (Ref.)	<0.001	1.00 (Ref.)	0.30	1.00 (Ref.)	<0.001	1.00 (Ref.)	0.39
Yes	3.94 (2.37-6.56)		2.43 (0.45-13.29)		2.68 (1.78-4.05)		1.55 (0.57-4.21)	
AJCC stage								
I	1.00 (Ref.)	<0.001	1.00 (Ref.)	0.08	1.00 (Ref.)	0.02	1.00 (Ref.)	0.12
II	0.47 (0.06-4.07)		1.00 (0.12-8.17)		0.75 (0.17-3.32)		0.62 (0.14-2.75)	
III	1.96 (0.27-14.24)		2.30 (0.31-16.86)		1.68 (0.41-6.87)		1.14 (0.28-4.70)	
Adjuvant chemotherapy								
No	1.00 (Ref.)	0.58	-		1.00 (Ref.)	0.47	-	
Yes	1.18 (0.67-2.07)		-		1.18 (0.75-1.83)		-	

OS: overall survival; HR: hazard ratio; CI: confidence interval; RFS: recurrence-free survival; Ref.: reference; AJCC: American Joint Committee on Cancer.

Dashes indicate variables not included in the multivariable model.

Variables with p<0.10 on univariable analysis were entered into the multivariable model.

### Survival outcomes

**Figure 1.**
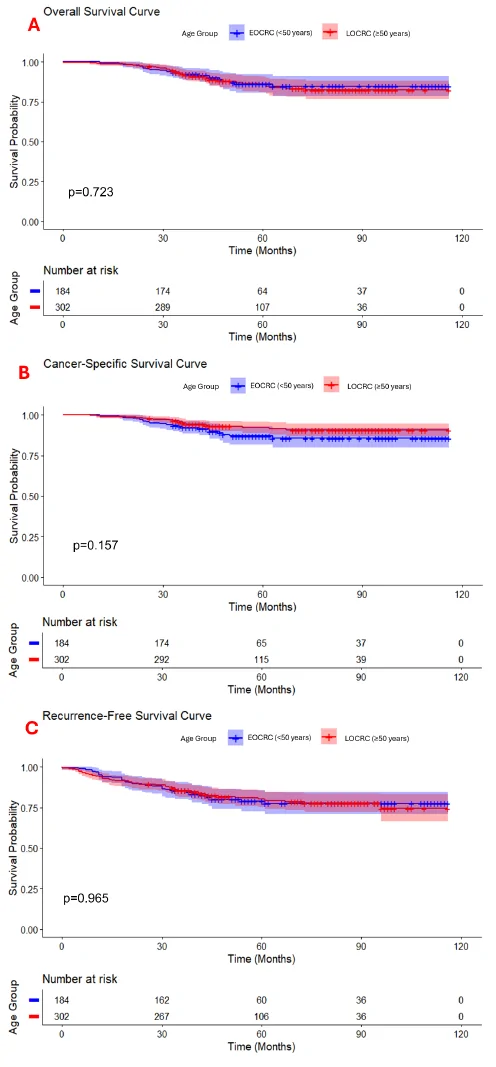
Kaplan-Meier estimates of survival outcomes by age in early- and late-onset colorectal cancer (EOCRC [<50 years] *vs*. LOCRC [≥50 years]). (A) Overall survival (OS; log-rank p=0.723). (B) Cancer-specific survival (CSS; log-rank p=0.157). (C) Recurrence-free survival (RFS; log-rank p=0.965). Numbers at risk are shown below each panel.

EOCRC: early-onset colorectal cancer; LOCRC: late-onset colorectal cancer.

Kaplan-Meier analysis of the overall cohort showed no statistically significant differences between EOCRC and LOCRC in OS, CSS, or RFS (Figure 1). The corresponding log-rank p-values were 0.723 for OS, 0.157 for CSS, and 0.965 for RFS, and the survival curves largely overlapped over the follow-up period.

**Figure 2.**
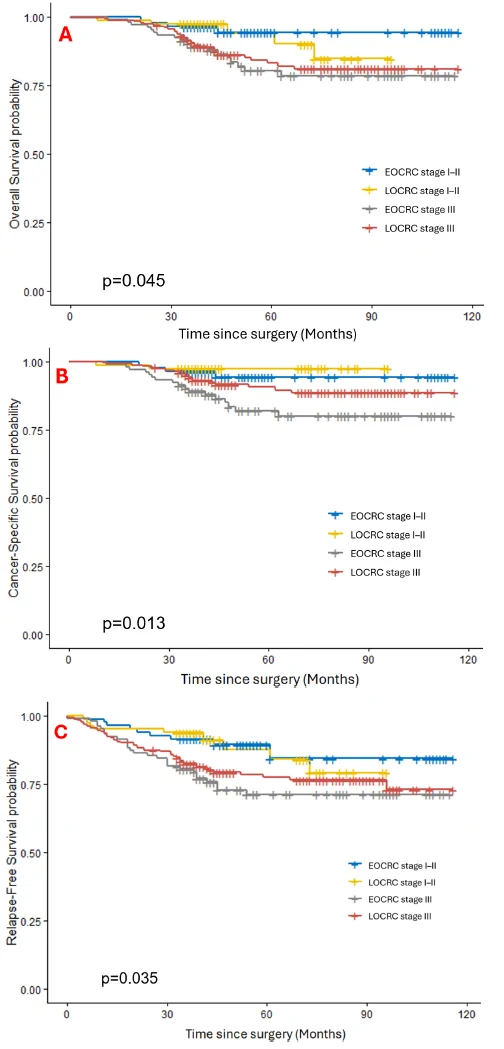
Stage-stratified Kaplan-Meier estimates of survival outcomes according to age at colorectal cancer onset and disease stage. Patients were stratified into four subgroups: EOCRC stage I-II, LOCRC stage I-II, EOCRC stage III, and LOCRC stage III. (A) Overall survival (global log-rank p=0.045). (B) Cancer-specific survival (global log-rank p=0.013). (C) Recurrence-free survival (global log-rank p=0.035).

EOCRC: early-onset colorectal cancer; LOCRC: late-onset colorectal cancer.

Stage-stratified Kaplan-Meier analysis demonstrated significant global differences among the four age-stage subgroups in OS (global log-rank p=0.045), CSS (global log-rank p=0.013), and RFS (global log-rank p=0.035) (Figure 2). However, these global comparisons do not directly establish EOCRC-versus-LOCRC differences within stage III disease. Stage-stratified curves suggested a less favorable survival pattern among patients with stage III EOCRC, particularly for CSS and RFS.

## DISCUSSION

In this single-center retrospective cohort of 486 patients undergoing curative resection for stage I-III colorectal adenocarcinoma in the Mekong Delta region of Vietnam, EOCRC was associated with a more aggressive histopathologic profile but similar survival outcomes compared with LOCRC in the overall cohort. Stage-stratified analyses suggested that survival differences related to age at onset may be more apparent among patients with stage III disease than among those with stage I-II disease. On multivariable analysis, postoperative complications were independently associated with worse outcomes, whereas age at onset was not.

The aggressive histopathologic profile observed in EOCRC in this cohort, including poorer differentiation, mucinous histology, and more frequent lymphovascular and perineural invasion, is consistent with findings from prior Asian and Western series, including reports by Park et al. and Carbone et al., in which younger patients with CRC more frequently exhibited poor differentiation, mucinous histology, and lymphovascular invasion compared with older counterparts^2^ ^,^ ^16^. Notably, the frequency of perineural invasion among EOCRC patients in this cohort (37.0%) exceeded that reported in most comparable series, and the overall burden of adverse histopathologic features may reflect delayed presentation in a setting without organized population-based screening, a persistent challenge in many low- and middle-income countries^4^ ^,^ ^6^ ^,^ ^7^ ^,^ ^19^. The greater receipt of adjuvant chemotherapy among EOCRC patients likely reflects both their more adverse histopathologic features and the greater likelihood that younger adults receive systemic treatment^5^ ^,^ ^8^ ^,^ ^14^ ^,^ ^20^. However, because adjuvant chemotherapy was not an independent prognostic factor in the multivariable model, its contribution to the observed survival equivalence between groups warrants further investigation.

The most clinically important finding of this study was the strong adverse prognostic association of postoperative complications. On multivariable analysis, postoperative complications were associated with more than an eight-fold increase in mortality risk and a marked increase in recurrence risk, underscoring that perioperative events may have a greater influence on long-term outcomes than age at onset alone. Although postoperative complications have been associated with worse survival across prior East Asian series, the hazard ratios reported for disease-free or cancer-specific survival in these studies ranged from approximately 1.6 to 3.0^9^ ^,^ ^10^ ^,^ ^11^ ^,^ ^12^, substantially lower than the estimates observed in the present cohort (HR 8.28 for OS; HR 4.81 for RFS). This discrepancy may partly reflect differences in complication definitions. Notably, Matsuda et al.^12^ examined infectious complications specifically, whereas the present analysis encompassed all postoperative complications regardless of type. The discrepancy may also reflect differences in perioperative infrastructure and complication management between a regional Vietnamese tertiary center and the high-volume institutions represented in the comparison cohorts. In this context, optimization of perioperative quality of care and surgical safety pathways may represent a modifiable and immediately relevant target for improving oncologic outcomes in this regional Vietnamese setting.

Disease stage remained an important determinant of prognosis in this cohort, consistent with prior studies showing that tumor burden is more closely associated with oncologic outcomes than age alone^4^ ^,^ ^5^ ^,^ ^7^ ^,^ ^8^. The attenuation of the prognostic effects of lymphovascular and perineural invasion after multivariable adjustment in prior reports likewise suggests that the impact of these adverse histopathologic features may be partly mediated through stage^4^. By contrast, the independent prognostic role of age at onset has been inconsistent across studies, with some cohorts reporting no association after adjustment and others showing worse outcomes only in selected high-risk subgroups^4^ ^,^ ^5^ ^,^ ^6^ ^,^ ^7^ ^,^ ^8^ ^,^ ^19^.

Age-related prognostic differences became apparent only after stratification by disease stage. Survival outcomes appeared largely similar between EOCRC and LOCRC in stage I-II disease, whereas the stage-stratified curves suggested a less favorable pattern for CSS and RFS among patients with stage III EOCRC. This stage-dependent pattern is consistent with the hypothesis that adverse histopathologic features, particularly lymphovascular and perineural invasion, are more prevalent among younger patients and may exert their prognostic influence most clearly in the setting of greater locoregional disease burden, where radical resection alone may be insufficient to offset tumor aggressiveness^4^ ^,^ ^20^. These findings align with the IDEA database analysis by Fontana et al.^5^ and SEER-based studies, both of which identified survival disadvantages associated with EOCRC primarily in higher-risk stage III subgroups^3^ ^,^ ^5^.

These findings have direct clinical implications for CRC management in Vietnam and similar Southeast Asian settings. The disproportionate burden of adverse histopathologic features among patients younger than 50 years, in a setting without organized population-based screening, supports heightened diagnostic attention to colorectal symptoms in younger adults and may add to the rationale for considering earlier screening strategies in selected populations^7^ ^,^ ^8^ ^,^ ^16^ ^,^ ^17^. In addition, the strong prognostic association of postoperative complications highlights perioperative quality improvement as a modifiable target for improving oncologic outcomes in regional surgical practice. Finally, because age at onset was not an independent prognostic factor in this cohort, postoperative surveillance and adjuvant treatment planning should be guided primarily by disease stage and adverse histopathologic features rather than by age alone^1^ ^,^ ^4^ ^,^ ^8^ ^,^ ^14^ ^,^ ^19^. In parallel, prevention of postoperative complications should be prioritized as a modifiable target for improving long-term oncologic outcomes.

Several limitations warrant consideration. First, this retrospective single-center cohort from a tertiary hospital in the Mekong Delta may have enriched the study population for patients with symptomatic or more advanced disease, particularly in a setting without organized population-based CRC screening. Therefore, the observed distributions of stage and adverse histopathologic features may not fully represent the broader CRC population in southern Vietnam. Second, the median follow-up of 47 months may be insufficient to capture late recurrence and longer-term survival differences, particularly among younger patients. Third, the absence of molecular profiling data limited the ability to assess biologic heterogeneity within EOCRC. In a prior study from the same institution, KRAS exon 2 mutations were identified in approximately one third of CRC patients^18^, suggesting that molecular characterization may be relevant to survival interpretation in this population and should be incorporated into future studies. Despite these limitations, this study provides clinically relevant real-world data from the Mekong Delta region, a Southeast Asian setting that remains underrepresented in the international CRC literature.

## CONCLUSIONS

EOCRC was characterized by a more aggressive histopathologic profile, but age-related prognostic differences became apparent only after stratification by disease stage, with postoperative complications and stage remaining the main determinants of outcome.

## Data Availability

The datasets generated and/or analyzed during the current study are available from the corresponding author upon reasonable request.
